# CD22 is required for formation of memory B cell precursors within germinal centers

**DOI:** 10.1371/journal.pone.0174661

**Published:** 2017-03-27

**Authors:** Craig P. Chappell, Kevin E. Draves, Edward A. Clark

**Affiliations:** 1 Department of Immunology, School of Medicine, University of Washington, Seattle, Washington, United States of America; 2 Department of Microbiology, School of Medicine, University of Washington, Seattle, Washington, United States of America; Jackson Laboratory, UNITED STATES

## Abstract

CD22 is a BCR co-receptor that regulates B cell signaling, proliferation and survival and is required for T cell-independent Ab responses. To investigate the role of CD22 during T cell-dependent (TD) Ab responses and memory B cell formation, we analyzed Ag-specific B cell responses generated by wild-type (WT) or CD22^-/-^ B cells following immunization with a TD Ag. CD22^-/-^ B cells mounted normal early Ab responses yet failed to generate either memory B cells or long-lived plasma cells, whereas WT B cells formed both populations. Surprisingly, B cell expansion and germinal center (GC) differentiation were comparable between WT and CD22^-/-^ B cells. CD22^-/-^ B cells, however, were significantly less capable of generating a population of CXCR4^hi^CD38^hi^ GC B cells, which we propose represent memory B cell precursors within GCs. These results demonstrate a novel role for CD22 during TD humoral responses evident during primary GC formation and underscore that CD22 functions not only during B cell maturation but also during responses to both TD and T cell-independent antigens.

## Introduction

The B cell-associated receptor, CD22, binds to alpha 2,6-galactose-linked sialic acids that are widely expressed throughout the body. CD22 has a number of ascribed functions including inhibition of BCR signaling via recruitment of SHP-1 phosphatase, as well as facilitation of adhesion between B cells and other cell types [1]. CD22 also regulates B cell homeostasis, survival and migration, and dampens TLR and CD40 signaling [2–4] CD22-deficient (CD22^-/-^) mice have reduced numbers of splenic marginal zone B cells [5,6] and display defective antibody (Ab) responses to T cell-independent (TI) antigens (Ags) [6–8].

It remains unclear to what extent CD22 regulates the development of T cell-dependent (TD) Ab responses and memory B cell formation. Initial studies from our lab and others concluded that CD22^-/-^ mice have normal responses to TD Ags [6–8]; however, mice were evaluated for only up to 35 days following immunization, and secondary Ag challenges were administered before primary immune responses had subsided. Ligands for CD22 have been identified on CD22 itself, IgM, and on T cells [9–11]. CD22 engagement in *trans* with CD22 ligands on T cells may regulate T cell activation [12,13]. Mice unable to express CD22 ligands (ST6GalI^-/-^ mice) have normal T cells but defective TD Ab responses to Ag + adjuvant or influenza infection [14,15]. Finally, in addition to inhibition of BCR signaling through surface IgM and IgD [6–8], CD22 also affects intracellular free calcium released by B cells expressing IgG [16,17]. Thus, multiple possibilities exist where CD22-CD22L interactions may influence TD B cell responses.

To further investigate the role of CD22 in TD Ab responses and memory B cell formation, we crossed CD22^-/-^ mice with B1-8^hi^ knockin mice expressing a VH gene that, when paired with a lambda1 L chain, generates a BCR with high affinity for the hapten, 4(hydroxy-3-nitrophenyl)acetyl (NP) [18]. Although CD22^-/-^ B1-8^hi^ B cells were able to respond to immunization with TD Ag and develop into germinal center (GC) B cells, they were not able to differentiate efficiently into memory B cells or long-lived plasma cells (LLPCs) and did not sustain Ab levels over time. We found that the lack of GC output correlated with a failure of CD22^-/-^ B cells to develop a subset of GC B cells delineated by CXCR4 and CD38 expression. These results suggest that CD22 plays an important role during TD Ab responses to generate a subset of GC B cells that may represent GC-derived precursors of memory B cells and LLPCs.

## Results and discussion

**Fig 1:**
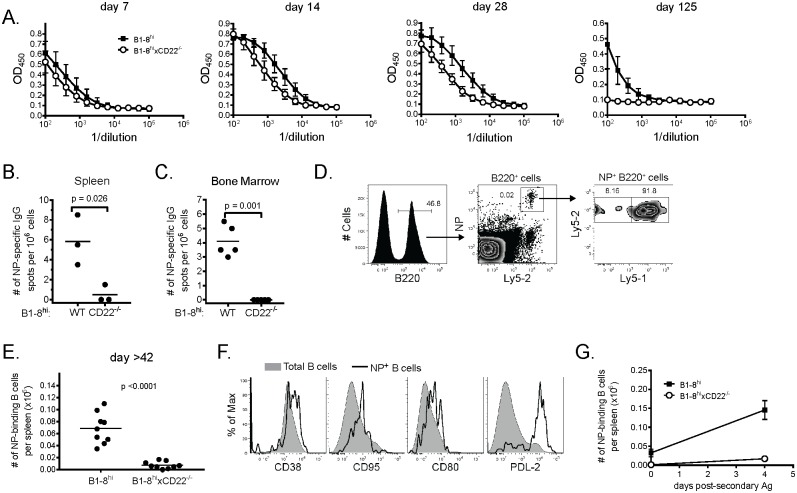
CD22^-/-^ B cells mount normal early TD Ab responses but do not form memory B cells or long-lived plasma cells. Splenocytes from WT or CD22^-/-^ B1-8^hi^ mice containing 2 x 10^5^ NP-specific B cells were adoptively transferred to B6 recipients 24 h prior to immunization with 50 micrograms NP-CGG in alum. (*A*) ELISA analysis of sera from WT (black squares) or CD22^-/-^ (white circles) recipients measuring anti-NP IgG1^a^ Abs on the indicated days. Data are from 3–5 mice per group per time point and are representative of 2 independent experiments. (*B*,*C*) The frequency of AFCs secreting NP-specific IgG1 Ab from spleen (*B*) or bone marrow (*C*) as determined by ELISPOT are plotted. Each dot represents a single animal with the mean indicated by a horizontal line. For *D*-*G*, splenoctyes containing 2 x 10^5^ NP-specific B cells from both WT and CD22^-/-^ B1-8^hi^ mice were adoptively transferred to Ly5.1 recipient animals that were then immunized as in *A*. (*D*) Representative flow cytometry analysis identifying NP-specific splenic B cells 125 days p.i. (*E*) Total number of NP-specific splenic B cells on day 42, 60 and 125 p.i. Each dot represents a single animal with the mean indicated by a horizontal line. 3 animals were used per time point. (*F*) Surface expression of the indicated marker was determined on total splenic B cells (shaded histogram) or WT B1-8^hi^ B cells (black line) from recipient mice immunized 125 days previously. Data are representative of 3 independent experiments using 3 mice per group. (*G*) Mean number of NP-specific B cells +/- SEM are plotted for immune mice (day 125 p.i.) before and 4 days after re-challenge with 20 micrograms soluble NP-CGG. One of two independent experiments using 3–4 mice/group is shown.

Previous studies have reported that CD22^-/-^ mice mount normal primary Ab responses to TD antigens [6–8], yet establishment of long-term humoral immunity was not reported. To assess if CD22-deficient B cells were capable of undergoing the steps that normally occur during responses after TD immunization, we transferred splenocytes from WT or CD22^-/-^ B1-8^hi^ mice into individual WT B6 recipients, immunized them 24 h later with NP-CGG in alum and analyzed IgG1^a^ (to distinguish Ab produced by transferred cells) anti-NP Ab responses over time. CD22^-/-^ B cells mounted anti-NP IgG1 Ab responses that were initially comparable to those of WT B cells (Fig 1A). However, serum Ab responses generated by CD22^-/-^ B cells steadily decreased after day 7 p.i. and became undetectable by 125 days p.i., whereas Ab from WT B cells remained detectable. Analysis of NP-specific LLPCs by ELISPOT in both spleen (Fig 1B) and bone marrow (Fig 1C) 42 days p.i. revealed a significant decrease in the number of LLPCs in mice that received CD22^-/-^ B cells compared to WT B cells.

To determine whether memory B cell formation was similarly affected by CD22-deficiency in B cells, we co-transferred WT Ly5.1^+^Ly5.2^+^ and CD22-deficient Ly5.1^-^Ly5.2^+^ B1-8^hi^ B cells into Ly5.1^+^ B6 recipients and immunized as above. Using Ly5 (CD45) expression to discriminate between WT and CD22^-/-^ B cells by flow cytometry, we found that ~90% of B220^+^ NP-binding B cells were derived from Ly5.1^+^Ly5.2^+^ WT B cells 125 p.i. (Fig 1D). Similar results were seen at day 42 and 60 p.i. (Fig 1E). We obtained similar results when WT and CD22^-/-^ B cells were transferred into separate recipients. WT B cells detected at these later time points were indeed memory B cells because they expressed multiple surface markers associated with memory B cells (CD38, CD80, CD95 and PD-L2) (Fig 1F) [19] and rapidly expanded upon re-challenge with soluble Ag (Fig 1G) (data are from re-challenge at 125 days p.i). We conclude from these results that CD22-deficient B cells were unable to populate the memory B cell or LLPC pools under the conditions tested.

**Fig 2:**
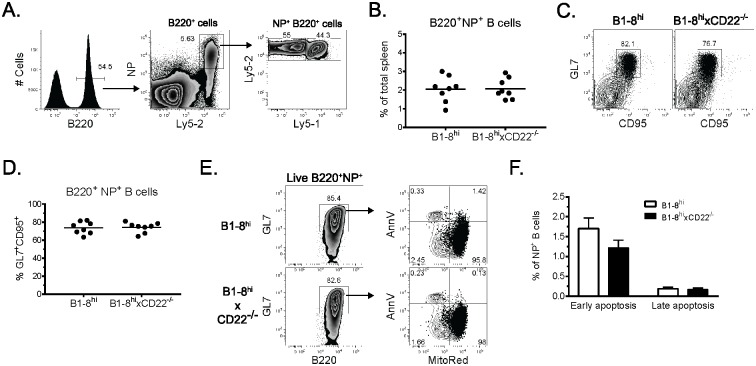
CD22-deficient B cells expand normally and undergo germinal center differentiation. Adoptive transfers and immunizations were performed as in Fig 1A/1B. (A) Representative flow cytometry analysis showing the frequency of WT and CD22^-/-^ splenic B1-8^hi^ B cells 7 days p.i. Data from 3 independent experiments are summarized in (*B*). Each dot represents a single animal with the mean indicated by a horizontal line. (*C*) Representative flow cytometric plots show the frequency of NP-specific splenic B cells (gated as in *A*) that co-express GL7 and CD95 7 days p.i. Data from 3 independent experiments are summarized in (*D*) where each dot represents a single animal with the mean indicated by a horizontal line. (*E*) NP-specific B cells were assessed for cell death. Representative flow cytometry plots depict Annexin V binding and Mitotracker Red exclusion among live NP-specific GL7^+^ splenic B cells identified as in (*A*) 7 days p.i. (*F*) Summarized data from one of 3 independent experiments using 3 mice per group.

CD22-deficient B cells have decreased survival compared to their WT counterparts [8]. Although CD22^-/-^ B cells mounted normal early TD Ab responses, it was possible that decreased survival prevented extensive B cell expansion and GC differentiation. To examine these possibilities, co-transfers and immunizations were performed as above and NP-binding B cells were analyzed by flow cytometry 7 days p.i. Both WT and CD22^-/-^ NP-binding B cells expanded to similar frequencies (Fig 2A and 2B). Surprisingly, we also observed similar frequencies of WT and CD22^-/-^ NP-binding B cells that expressed the GC-associated markers GL7 and CD95 (Fig 2C and 2D). Lastly, we found similar frequencies of apoptotic cells among NP^+^ GL7^+^ CD22^-/-^ and WT B cells 7 days p.i. (Fig 2E and 2F). These results suggest the defect in CD22^-/-^ B cells is not due to decreased survival or a failure to develop GCs. We conclude that CD22^-/-^ B cells undergo expansion and GC differentiation comparable to WT B cells.

**Fig 3:**
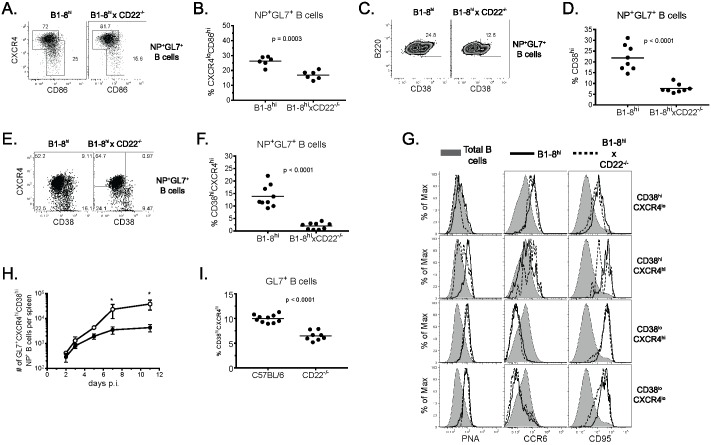
CD22-deficient B cells fail to form CD38^hi^CXCR4^hi^ GC B cells. Adoptive transfers and immunizations were performed as in Fig 1. (*A*) Representative flow cytometric plots show CXCR4 and CD86 expression among NP-specific GL7^+^ splenic B cells gated as in Fig 2A 7 days p.i. (*B*) Summary data from 2 independent experiments using 3 mice per group showing the frequency of NP-specific GL7^+^ LZ (CXCR4^lo^CD86^hi^) B cells 7 days p.i. Each dot represents an individual animal. (*C*,*E*) Representative flow plots show the frequency of cells expressing CD38 (*C*) or CD38 and CXCR4 (*E*) among NP-specific GL7^+^ B cells 7 days p.i. (*D*,*F*) Summary data of CD38 (*C*) or CD38 and CXCR4 expression (*E*) from 3 independent experiments using 2–3 mice per group. Each dot represents an individual animal. (*G*) Histograms show PNA, CCR6 or CD95 expression on each population (P1-P4) of NP-specific GL7^+^ splenic B cells depicted in the diagram at left. Total (shaded), WT (solid line) and CD22^-/-^ (dashed line) B cells are shown. Data are representative of 2 independent experiments using 5–7 mice each. (*H*) Frequencies of WT (white circles) and CD22-/- (black circles) NP-specific GL7^+^CD38^hi^CXCR4^hi^ splenic B cells are plotted over time. Data are from one experiment utilizing 7 mice/group at each time point. (*I*) Summary data depicting the frequency of CD38^hi^CXCR4^hi^ GC B cells in WT and CD22^-/-^ mice (*E*) from 2 independent experiments using 4–6 mice per group. Each dot represents an individual animal.

The fact that CD22^-/-^ B cells underwent apparent normal GC differentiation yet failed to generate memory B cells or LLPCs implies that selection or development of memory B cell precursors within GCs may be dysregulated in the absence of CD22. The prevailing model of selection within GCs encompasses cyclic recycling whereby GC B cells undergo reiterative CXCR4-dependent cycling between GC light zones (LZ), where they acquire Ag from FDCs, and dark zones (DZ), where they undergo proliferation and somatic hypermutation. To investigate GC sub-populations more closely, we analyzed GC B cells 7 days p.i. for CXCR4 and CD86 expression to determine if CD22-deficient B cells were capable of forming both DZ and LZ B cells. We included CD38 in the analysis as an additional marker of B cell subsets since CD38 is found on naïve B cells, early GC precursors and memory B cells, but not GC B cells [20]. Among NP^+^ GL7^+^ B cells, those deficient in CD22 formed slightly lower but significant (p<0.001) frequencies of CXCR4^lo^CD86^hi^ LZ GC B cells compared to WT B cells (Fig 3A and 3B). This trend was evident at day 14 p.i. but was not statistically significant. Analysis of CD38 expression on day 7 p.i. revealed that a large fraction (15–30%) of WT GL7^+^ B cells co-expressed CD38 (Fig 3C and 3D). Interestingly, CD22^-/-^ B cells had significantly decreased frequencies of GL7^+^CD38^hi^ cells compared to WT B cells (p<0.0001). To further analyze the CD38^hi^ fraction, we examined co-expression of CXCR4 to determine if the decreased CD38^hi^ subset corresponded to the decrease in LZ B cells noted above. This analysis revealed that WT NP^+^ GL7^+^ B cells could be subdivided into 4 populations based on CXCR4 and CD38 expression (Fig 3E). Surprisingly, CD22^-/-^ GL7^+^ B cells had a highly selective and significant decrease (p < 0.0001) in cells co-expressing CXCR4 and CD38 (Fig 3F).

Previous studies have suggested GL7^+^CD38^hi^ B cells represent pre- or very early GC B cells that give rise to both GC-dependent and GC-independent memory B cells [20, 21]. To determine which subsets of NP-specific GL7^+^ B cells possessed a GC phenotype, we analyzed each subpopulation of NP-binding GL7^+^ B cells (CXCR4^lo^CD38^hi^, CXCR4^hi^CD38^hi^, CXCR4^hi^CD38^lo^, CXCR4^lo^CD38^lo^) for expression of PNA, CD95, and CCR6 on day 7 p.i. (Fig 3G). CXCR4^lo^CD38^hi^ B cells displayed low levels of PNA binding, high levels of CCR6, and elevated CD95 expression, indicating these cells were activated but not GC B cells. In contrast, CXCR4^hi^CD38^hi^ B cells expressed high levels of PNA binding and high levels of both CCR6 and CD95 expression. This phenotype suggests that CXCR4^hi^CD38^hi^ B cells are bona fide GC B cells as PNA binding is the universal standard for GC B cell identification. Analysis of the two CD38^lo^ subsets showed both CXCR4^lo^ and CXCR4^hi^ populations had the prototypical GC phenotype: high PNA binding, low CCR6 expression, and high CD95 expression. CXCR4^hi^CD38^hi^ WT GC B cells began to appear on day 3 p.i. and slowly increased over time, whereas CD22-deficient B cells did not significantly develop this population and thus remained consistently fewer in number than WT CXCR4^hi^CD38^hi^ GC B cells (Fig 3H). We conclude that CD38 and CXCR4 expression can be combined to discriminate 4 populations of activated (i.e., GL7^+^) B cells, and that CD22 plays an important role in the formation of a population of GL7^+^CXCR4^hi^CD38^hi^PNA^hi^ GC B cells whose absence correlates with an inability to form memory B cells and LLPCs.

To assess whether the reduction of GL7^+^CXCR4^hi^CD38^hi^PNA^hi^ CD22^-/-^ GC B cells might be due to an inability of CD22^-/-^ B cells to compete with WT B cells at some point during GC formation, we compared GC B cell formation in CD22^-/-^ mice to WT mice after immunization with NP-CGG and alum. As seen in the adoptive transfer system, NP-specific IgG Ab responses were slightly lower in CD22^-/-^ mice while total GL7^+^ B cells levels were normal 7 days p.i. Furthermore, immunized CD22^-/-^ mice had a significantly decreased frequency of GL7^+^CXCR4^hi^CD38^hi^ GC B cells (Fig 3I, p<0.0001). Thus, even in the absence of WT B cells, CD22^-/-^ B cells had an intrinsic defect manifest at this stage of GC formation. However, the deficiency in CD22^-/-^ mice was not as pronounced as seen in the adoptive transfer system (Fig 3F), suggesting that CD22^-/-^ GC B cells may not be able to compete well with WT B cells as they develop into the CXCR4^hi^CD38^hi^ subset.

**Fig 4:**
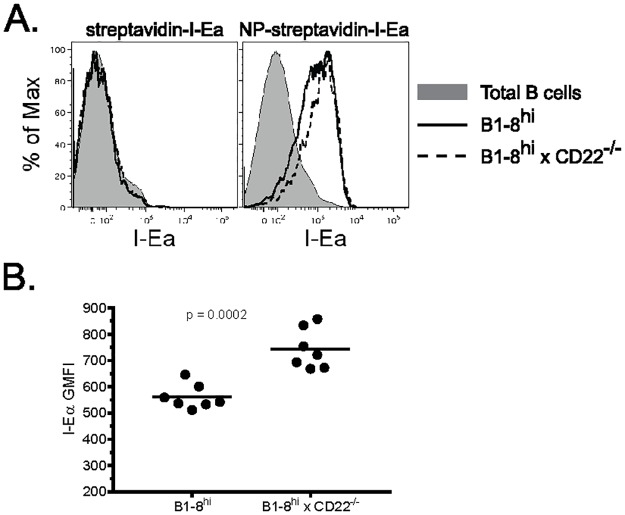
CD22-deficient GC B cells are capable of Ag processing and presentation. Recipient mice were immunized with 50 micrograms NP-CGG and 6 days later injected i.v. with 5 micrograms NP-streptavidin-I-Ealpha or streptavidin-I-Ealpha. 18 h later mice were sacrificed and spleens analyzed by flow cytometry. (*A*) Representative analysis of peptide:MHCII expression among NP-specific GL7^+^ WT (solid lines) and CD22^-/-^ (dashed lines) B cells. Total B220^+^ cells are depicted by shaded histograms for reference. (*B*) Summarized data from experiment in (*A*). Each dot represents a single animal with the mean indicated by a horizontal line. Data are representative of 2 independent experiments using 3–7 mice each.

Ag presentation to CD4 T_FH_ cells within LZs is required for GC B cells to differentiate to either memory B cells or LLPCs. The failure of CD22-deficient B cells to develop a CXCR4^hi^CD38^hi^ subset, thus, may be due to an inability to present Ag to CD4 T_FH_ cells. To test this, we co-transferred WT and CD22^-/-^ splenocytes into recipient mice as above and injected them 6 days p.i. with NP-coupled streptavidin, to which biotinylated I-Ealpha peptide was bound (NP-streptavidin-I-Ealpha). The I-Ealpha peptide, when presented in the context of I-A^b^ is recognized by the Y-Ae mAb [22]. One day later the mice were sacrificed and NP-specific GL7^+^ B cell subsets (based on CXCR4 and CD38 expression) were analyzed for Ag presentation by flow cytometry using the Y-Ae mAb. Surprisingly, NP-specific GL7^+^ CD22^-/-^ B cells if anything were more efficient in presenting pMHCII than their WT counterparts (Fig 4A and 4B). Ag presentation required uptake via Ag-specific BCRs because neither non-specific B cells (total B220^+^) nor NP-specific GL7^+^ B cells from mice that received streptavidin-I-Ealpha (without NP) displayed pMHCII (Fig 4A). All four populations of GL7^+^ NP-specific B cells expressed pMHCII, demonstrating that CD22-deficient GC B cells are fully capable of Ag capture, processing and presentation in the presence of WT B cells at this stage. We conclude from these experiments that competition with WT B cells for Ag capture is not a factor contributing to their reduced numbers.

In contrast to our results, a previous study (Onodera et al.) [23] reported that after immunization CD22^-/-^ B cells, including GC B cells, rapidly expand and generate short-lived AFCs and antibodies. Unlike in our study, the recipient mice used by these investigators were previously immunized with CGG (‘carrier primed’). Thus, both CGG-specific Tfh cells and CGG-anti-CGG immune complexes that can be efficiently taken up by FcγR^+^ cells may have contributed to the rapid hyperproliferative and extrafollicular B cell responses they observed, as has been reported [24]. Such hyperproliferation was clearly not evident in the bona fide primary immune responses we measured. In our studies, numbers of total Ag-specific WT and CD22^-/-^ B cells did not significantly differ. Nevertheless, our results support the conclusion of Onodera et al. [23] that CD22^-/-^ B cells do not efficiently generate memory B cells. Our results suggest that the defect in CD22^-/-^ B cells was first evident during early GC formation, and that CD22 was important for forming a population of GL7^+^CXCR4^hi^CD38^hi^PNA^hi^ GC B cells whose deficiency was associated with a lack of memory B cells and LLPCs.

CD22 regulates BCR signaling chiefly via recruitment of the SHP-1 phosphatase. Two groups have reported a critical role for SHP-1 in GC maintenance and memory cell development [25, 26]. Although our studies here do not address SHP-1 recruitment in CD22^-/-^ GC B cells, they are consistent with the notion that the absence of CD22 leads to decreased SHP-1 recruitment. Unlike SHP-1 deletion, however, GCs are not ablated in the absence of CD22; rather, a small subset of PNA^+^ GC B cells denoted by CXCR4 and CD38 expression fail to develop. These cells appeared early in the immune response (day 3) and accounted for approximately 10% of the total GL7^+^ B cell population until day 14. Thus, it appears CD22 is required for the generation of this early subset. Cognate interactions between B and T cells are critical for GC initiation and maintenance, and CD22 ligands are expressed on T cells as well as B cells [11,27]. Interestingly, high affinity ligands for CD22, while present on naïve and memory B cells, are lost on GC B cells [28]. Thus, one attractive possibility is that once CD22 is unmasked on GC B cells, CD22L-CD22 interactions may then occur *in trans* between CD22L^+^ CD4 T_FH_ cells and CD22^+^ GC B cells to promote further B cell survival and maturation. CD22^-/-^ GC B cells that are not capable of receiving this type of ‘help’ from T_FH_ cells may not be as competent as WT B cells for memory B cell formation. Thus, in addition to altered BCR signaling, defective interactions between B and T cells may also contribute to the lack of memory formation by CD22^-/-^ B cells. Further studies will be required to discriminate between these possibilities.

## Materials and methods

### Mice

Ly5.2^+^ and Ly5.1^+^ C57BL/6 (B6) mice were bred and maintained by our laboratory or alternatively purchased from The Jackson Laboratory. Ly5.1^+^ B1-8^hi^ mice [18] were kindly provided by Dr. Michel Nussenzweig (Rockefeller University, New York, NY) and crossed to Ly5.2^+^ B6 mice to generate Ly5.1^+^ Ly5.2^+^ B1-8^hi^ animals. CD22^-/-^ mice on a Ly5.2^+^ B6 background were prepared as described [8]. Ly5.1^+^ B1-8^hi^ mice were backcrossed to CD22^-/-^ mice to obtain Ly5.2^+^ CD22^-/-^ B1-8^hi^ animals. All mice (8–15 weeks of age) were maintained under specific pathogen-free conditions and used in accordance with and approval by the University of Washington Institutional Animal Care and Use Committee guidelines.

### Adoptive transfers and immunizations

Splenocytes from Ly5.1^+^ Ly5.2^+^ B1-8^hi^ or Ly5.2^+^ CD22^-/-^ B1-8^hi^ mice containing 2 x 10^5^ NP-specific B cells were transferred i.v. to either Ly5.1^+^ or Ly5.2^+^ B6 recipients as indicated 24 h prior to i.p. immunization with 50 micrograms NP-CGG precipitated in alum. In some experiments mice were boosted with 20 micrograms soluble NP-CGG in PBS (i.v.). Total spleen cell counts were obtained using a hemocytometer. Frequencies of NP-specific B cells were obtained by flow cytometry for NP-binding B cells.

### Reagents

3(hydroxy-4-nitrophenyl)acetyl (NP) (BioSource) was coupled to phycoerythrin (PE), chicken gamma globulin (CGG) or streptavidin (all from Sigma) as we previously described [29]. Biotinylated I-Ealpha peptide was synthesized by Invitrogen and used to generate both NP-streptavidin-I-Ealpha and NP-streptavidin-AF647-I-Ealpha which were prepared and tested in control experiments as described [28].

### Flow cytometry

The following Abs were used to stain splenocytes for flow cytometry: B220-BrilliantViolet(BV)605 (BioLegend), Ly5.2-AlexaFluor(AF)700, Ly5.1-PE-Cy7, GL7-APC, CD95-biotin, CCR6-biotin, CD38-FITC, CXCR4-PerCP-eFluor710, CD80-FITC, PD-L2-APC, and Y-Ae-biotin (all from eBioscience). Biotinylated Abs were detected with streptavidin-coupled BV421 (BioLegend). Additional reagents used for flow cytometry included PNA-FITC and NP-PE. Surface staining was carried out as described [29]. MitoTracker Red (Invitrogen), Annexin V-FITC (eBioscience) and SYTOX Red viability dye (Invitrogen) were used according to manufacturer’s instructions. Data were acquired on a LSRII flow cytometer (BD Biosciences) and analyzed using FlowJo software (Treestar, Inc.).

### ELISA and ELISPOT

Biotinylated anti-IgG1^a^ (Southern Biotech, Inc.) was used to distinguish Ab produced by transferred B1-8^hi^ B cells from Ab produced by endogenous B cells (IgG1^b^). ELISA and ELISPOT procedures were performed as previously described [30] with the exception that standard curves were not generated.

## Abbreviations

AFC: antibody-forming cell
B6: C57BL/6
CGG: chicken gamma globulin
DZ: dark zone
GC: germinal center
LLPC: long-lived plasma cell
LZ: light zone
NP: 4(hydroxy-3-nitrophenyl)acetyl
pMHCII: peptide:MHCII
TD: T cell-dependent
TI: T cell-independent
WT: wild-type
